# RISK OF BLEEDING COMPLICATIONS IN PERCUTANEOUS BILIARY DRAINAGE: THE PARADOX OF THE NORMAL HEMOSTASIS

**DOI:** 10.1590/0102-672020190001e1454

**Published:** 2019-10-21

**Authors:** Eduardo Javier HOUGHTON, Emilio INVERNIZZI, Pablo ACQUAFRESCA, Mariano PALERMO, Mariano E. GIMÉNEZ

**Affiliations:** 1 DAICIM Foundation; 2 Hospital Bernardino Rivadavia; 3 Universidad de Buenos Aires, Buenos Aires, Argentina.

**Keywords:** Biliary tract surgical procedures, Percutaneous biliary drainage, Hemobilia, Postoperative complications, Procedimentos cirúrgicos no trato biliar, Drenagem biliar percutânea, Hemobilia Complicações pós-operatórias

## Abstract

**Background:**

Percutaneous biliary drainage is a safe procedure. The risk of bleeding complications is acceptable. Frequently, patients with biliary obstructions usually have coagulation disorders thus increasing risk of bleeding. For this reason, patients should always fit the parameters of hemostasis.

**Aim:**

To determine whether the percentage of bleeding complications in percutaneous biliary drainage is greater in adults with corrected hemostasis prior to the procedure regarding those who did not require any.

**>Methods:**

*:* Prospective, observational, transversal, comparative by independent samples (unpaired comparison). Eighty-two patients with percutaneous biliary drainage were included. The average age was 64±16 years (20-92) being 38 male and 44 female. Patients who presented altered hemostasis were corrected and the presence of bleeding complications was evaluated with laboratory and ultrasound.

**Results:**

Of 82 patients, 23 needed correction of hemostasis. The approaches performed were: 41 right, 30 left and 11 bilateral. The amount of punctures on average was 3±2. There were 13 (15.8%) bleeding complications, 12 (20%) in uncorrected and only one (4.34%) in the corrected group with no statistical difference. There were no differences in side, number of punctures and type of drainage, but number of passes and the size of drainage on the right side were different. There was no related mortality.

**Conclusion:**

Bleeding complications in patients requiring hemostasis correction for a percutaneous biliary drainage was not greater than in those who did not require any.

## RESUMO

**Racional:**

A drenagem biliar percutânea é procedimento seguro. O risco de complicações hemorrágicas é aceitável. Frequentemente, os pacientes com obstruções biliares apresentam distúrbios de coagulação, aumentando o risco de sangramento. Por esse motivo, eles devem sempre ser adequados aos parâmetros da hemostasia.

**Objetivo:**

Determinar se a porcentagem de complicações hemorrágicas na drenagem biliar percutânea é maior em adultos com hemostasia corrigida antes do procedimento em relação àqueles que necessitaram nenhuma.

**Métodos:**

Estudo prospectivo, observacional, transversal, comparativo por amostras independentes (comparação não pareada). Oitenta e dois pacientes foram submetidos à drenagem biliar percutânea. A idade média foi de 64±16 anos (20-92), 38 eram homens e 44 mulheres. Os pacientes que apresentaram hemostasia alterada foram corrigidos, e a presença de complicações hemorrágicas foi avaliada com exames laboratoriais e ultrassonográficos.

**Resultados:**

Dos 82 pacientes, 23 necessitaram de correção da hemostasia. O acesso à direita foi em 41 casos, 30 à esquerda e 11 bilaterais. A quantidade de punções em média foi de 3±2. Houve 13 (15,8%) complicações hemorrágicas, 12 (20%) no grupo não corrigido e apenas uma (4,34%) no corrigido sem diferença estatística. Não houve diferenças no lado, no número de perfurações e no tipo de drenagem, mas o número de passagens e o tamanho da drenagem no lado direito foram diferentes. Não houve mortalidade.

**Conclusão:**

As complicações hemorrágicas em pacientes que necessitam de correção da hemostasia antes da drenagem biliar percutânea não são maiores do que naqueles que não a requerem.

## INTRODUCTION

Percutaneous biliary drainage (PBD) is a safe procedure for the treatment of biliary obstructions. It can be performed under ultrasound, fluoroscopic equipment and virtual or augmented reality guide^10^ ^,^ ^11^ ^,^ ^25^ ^,^ ^28^. Access may be right, left or bilateral^2^ ^,^ ^20^ ^,^ ^24^.

The causes can be benign or malignant. Within the first one are cholelithiasis, congenital stenosis, cystic dilations, surgical lesions of biliary tract, acute cholangitis, among others^1^ ^,^ ^4^ ^,^ ^7^ ^,^ ^9^ ^,^ ^14^ ^,^ ^15^ ^,^ ^18^ ^,^ ^19^ ^,^ ^23^ ^,^ ^24^. The malignant biliary obstruction is caused by primary or secondary tumors^1^ ^,^ ^4^ ^-^ ^9^ ^,^ ^19^ ^,^ ^24^ ^,^ ^28^.

There are several complications described in the literature^21^ ^,^ ^22^ ^,^ ^24^, and bleeding is one of them. It has a wide range of severity from asymptomatic to major bleeding that can endanger the patient’s life. The rate of bleeding complications can range from 3-26% depending on the series, being hematomas, hemobilia, hemoperitoneum, arteriovenous and bilioportal fistulas, and hemothorax^2^ ^,^ ^9^ ^,^ ^11^ ^,^ ^12^ ^,^ ^17^ ^,^ ^20^ ^,^ ^24^ ^,^ ^27^.

Frequently, patients with biliary obstructions have coagulation disorders, thus increasing risk of bleeding. According to the guidelines for the perioperative management of coagulation status and bleeding risk^30^, the PBD is considered high risk, and patients should always fit the parameters of hemostasis prior to the procedure. Therefore, the question of whether the correction of coagulation is sufficient to avoid bleeding complications arises.

 Thus, the aim of this study was to determine whether the percentage of bleeding complications in PBD is greater in patients with impaired hemostasis who get their coagulation corrected compared to those without disorders. 

## METHODS

All procedures performed in this study were in accordance with the ethical standards of the institutional and/or national research committee and with the 1964 Helsinki declaration and its later amendments or comparable ethical standards. Those who agreed to participate previously had to sign the Informed Consent and received Information Sheet The confidentiality of personal information recorded in medical history (or registration form or questionnaire or form), was respected. The researchers obeyed the terms of Law 1845 (Law of protection of personal data in force in the City of Buenos Aires).

It was followed the CONSORT and STROBE guidelines and checklist^31^ ^,^ ^32^ for the study design. This is a prospective, observational, transversal, comparative study by independent samples (unpaired comparison). It was realized from July 2015 to June 2016 in Rivadavia Hospital and DAICIM Foundation (Teaching, Assistance, Research Minimally Invasive Surgery), Buenos Aires, Argentina.

The inclusion criteria were: adult over 18 years in whom a PBD was performed for any cause. The exclusion criteria were: those patients who have undergone biliary drainage either percutaneous or endoscopic previously up to a week before referred by the patient or recorded in medical history and the ones who did not want to participate in the study. Elimination criteria were for the ones who died within 72 h after biliary drainage by other causes unrelated to bleeding complications; have undergone other abdominal procedure within 72 h after biliary drainage, and wanted to withdraw from the study. 

Sample size was considered by 10x30 number of variables plus 30 cases giving a total of 90 cases^3^.

Before the intervention, patients enrolled entered the operating room after performing a surgical risk, routine tests (blood count, blood glucose, uremia, coagulogram) and pre-anesthetic evaluation and signing the informed consent. Those with altered hemostasis were corrected before the procedure either with vitamin K, fresh frozen plasma (FFP), protamin or coagulation factors lyophilized concentrates in the doses indicated by hematology.

During the procedure, the anesthesiologist monitored continuously the hemodynamic status and vital signs with pulse oximetry, electrocardioscopy, capnography (if necessary), and non-invasive blood pressure. 

A medical team was responsible for collecting data in a printed form, while the operator performed the PBD.

The choice of puncture site was in charge of the operator. The technique involved puncturing the bile duct with a 21 G Chiba needle guided by ultrasound and/or fluoroscopy. Once inside the bile duct, a bile sample was taken for culture. A 0.018 guide wire was passed. D’Agostino introducer was placed and cholangiography was taken. A 0.035 hydrophilic wire was placed and a 4 Fr. Kumpe catheter tried to overcome stenosis. If achieved, biliary drainage with a multipurpose drain 8.5 or 10 Fr was placed and fixed to skin.

In the postoperative period, we monitored vital signs, control puncture site and control of immediate complications by performing an ultrasound at 24 h post-drainage. 

In case of occurrence of hematoma or hemoperitoneum without hemodynamic decompensation (defined as inability to maintain blood pressure without vasoactive drugs) conservative treatment must be carried out^27^. In case of hemodynamic decompensation and after its compensation, an eventual tactic was to perform exchange to a higher caliber catheter and arteriographic embolization done. In failure of these measures, laparoscopy or laparotomy was in consideration^27^.

When evident hemobilia through the catheter appeared, we proceeded to the quantification and used the same algorithm described above^27^.

### Variables studied

#### General

Cause of drainage, age in years, gender, bleeding complications (appearance of hematomas, hemobilia and/or hemoperitoneum diagnosed by ultrasound, CT, arteriography or fistulography through catheter in 24, 48 and/or 72 h post-drainage), coagulopathies correctly diagnosed (prothrombin concentration less than 70%, greater than 1.3 RIN and than 40 sec activated, partial thromboplastin time (aPTT under 100,000 platelets per mm) were annotated. 

#### Technical variables

The number of punctures (defined as the entry of the needle through Glisson capsule) and of passes (defined as the number of times the needle was removed and progressed without leaving the liver and therefore without piercing Glisson capsule again) made during PBD were observed. Puncture sites were right, left or both lobes of the liver following the Cantlie´s line anatomy. Additionally, number of drains and drain types (external or internal and external) were collected.

#### Laboratory findings

Serum bilirubin, serum albumin, hematocrit and hemoglobin, prothrombin concentration, aPTT, RIN, platelet count were measured. 

### Statistical analysis

The data were entered into a database (Microsoft Excel 97) and then were analyzed using the statistical package (SPSS Medcalc 19 and 14). Frequency distribution and/or percentages relative to the total cases were established for all variables. For measures in ordinal scale or higher, we described: number of cases, minimum value, maximum value, arithmetic mean, typical deviation and standard error. When necessary, confidence intervals of 95% (IC95) were estimated and we used as tests of significance, Student Test, Mann Whitney test, Chi square test, Fisher test. We set the level of significance at alpha 0.05.

## RESULTS

Data case number 3 was eliminated due to the cause of obstruction: gallblader lithiasis with choledocolithiasis, therefore an immediate laparoscopic cholecistectomy was carried out.

Data of the remaining 82 patients with different pathologies with biliary obstructions received PBD. The average age was 64±16 years (range=20, median=65, maximum=92).

Biliary obstruction causes were found as follows: periampullar tumors: 37%; high tumors: 28%; middle tumors, 9%; hepaticojejunostomy stenosis, 11%; lithiasis, 10%; benign stenosis, 6%. 

Female represented 53.7% (44/82) of the cases and male the rest. The average age among women was 60±19 vs. 68±13 among males. The differences between the mean age by gender were statistically significant (t=2.35, p=0.0213).

Puncture sites were 50% on the right, 36.6% of the left and the rest, bilateral (13.4%). The amount of punctures and passes on average wre 3±2 (minimum=1, medium=3, maximum=10) and 8±9 (minimun=1, medium=5, maximum=57) respectively. 

In 28% (23/82) of patients was required correction of hemostasis. Nine were corrected with fresh frozen plasma (FFP), six with platelet transfusion, five with coagulation factors lyophilized concentrates, one with protamin sulfate, 11 with vitamin K. Six patients received more than one type of correction, as follows: two FFP plus vitamin K; one FFP plus platelets plus coagulation factors lyophilized concentrates plus vitamin K; one FFP plus protamin plus vitamin K; one platelets plus vitamin K; one coagulation factors lyophilized concentrates plus vitamin K. 

Only 15.85% (13/82) had post-treatment bleeding complications and no deaths were associated with the procedure. 

We monitored the effect of confounding variables that could be related to the presence of bleeding complications before analyzing the variable under study “hemostasis correction”. We found no significant differences in the appearance of bleeding complications according to the mean age (Fisher p=0.487), between females and males (Fisher p=0.487), between malignant diseases and benign ones (Fisher p=0.45502). 

There were no statistically significant differences in the prevalence of complications according to puncture site (Table 1). However, we should increase sample size before making clinically applicable decisions (Chi^2^=5.25; p=0.072).

**TABLE 1:** Puncture site

Puncture site	Bleeding complications		Total
	Yes	No	
Right	5	33	38
	13%	87%	
Left	7	37	44
	16%	84%	
Bilateral	3	8	11
	27%	73%	
Total	12	70	82

**FIGURE 1:**
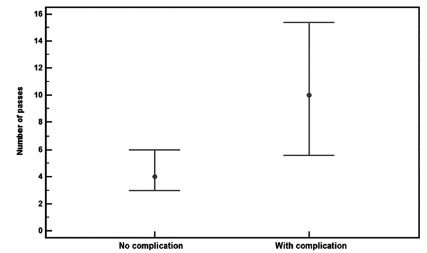
Median and confidence intervals 95% of number of passes in 82 patients with and without bleeding complications

The average number of punctures was similar in the group of patients with and without bleeding complications: 4 (1-6) and 3 (1-10) respectively (Mann-Whitney test Z=1.73; p=0.0824). The number of passes was significantly higher in those patients with hemorrhagic complications (Mann-Whitney test Z=2.33;p=0,0196, Figure 1). 

Mann-Whitney test Z=2.33;p=0,0196

**FIGURE 2:**
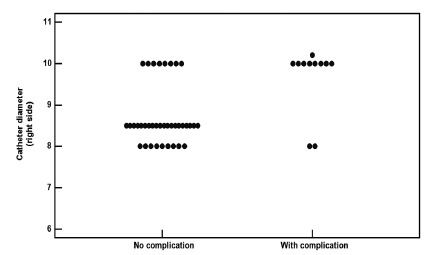
Dot plot: right catheter diameter in a 48 patients group with and without bleeding complications

The average diameter of the catheter in the right puncture site was significantly higher among the group of patients who had bleeding complications than in those who did not present this event (Figure 2). 

Mann-Whitney test Z=2.75; p=0.0059

The average diameter of the catheter in the left puncture site was 8.7 Fr. in the complicated group vs. 9.3 Fr in the non complicated group, similar in both who had bleeding complications and those who did not (Mann-Whitney test Z=1.42; p=0, 1557). 

There was no significant association between type of drainage and the presence of bleeding complications. However, the sample size should be increased before making clinically applicable decisions (Chi^2^=3.74; p=0.154, Table 2)

**TABLE 2:** Type of drainage

Type of drainage	Bleeding complications		Total
	Yes	No	
External	4	20	24
	17%	83%	
Internal	7	46	53
	13%	87%	
Both	2	2	4
	50%	50%	
Total	13	68	81

Regarding laboratory values performed in both groups of patients, only prothrombin concentration mean values were significantly higher in the group with bleeding complications than in those without such an event; however, both averages were above threshold value so they have no clinical significance. 

Finally in relation to the aim of the study, we could established that the number of bleeding complications in patients requiring hemostasis correction for a PBD was not higher than in those not requiring (Fisher p= 0,067 Table 3)

**TABLE 3:** Correction of hemostasis compare with bleeding complications

Hemostasis correction	Bleeding complications		Total
	YES	NO	
YES	1	22	23
	4%	96%	
NO	12	47	59
	20%	80%	
Total	13	69	82

On the other hand, we observed a trend to find more complications in the uncorrected group but we need to increase sample size to confirm this. Also, we decided to perform a separated analysis excluding patients who were corrected only with vitamin K, since vitamin K is simply a necessary co-factor for the synthesis of own patient’s coagulation factors, and therefore could be considered not as an external correction. Anyway, as shown in the Table 4, the results are similar and there is no association between bleeding complications and correction of coagulation (Fischer exact test p=0,132).

**TABLE 4:** Hemostasis correction excluding those corrected only with vitamin K

Hemostasis correction	Bleeding complications		Total
	Sim	No	
Yes	1	17	18
	6%	94%	
No	12	47	59
	20%	80%	
Total	13	64	77

Fischer exact test (p)=0,132

## DISCUSSION

Bleeding complications in PBD are relatively frequent. They include hematomas, hemobilia and even hemoperitoneum. Reported rates vary between 2% and 16%, most of them of low severity^2^ ^,^ ^9^ ^,^ ^11^ ^,^ ^12^ ^,^ ^17^ ^,^ ^20^ ^,^ ^24^ ^,^ ^27^. According to L’Hermineet^11^, they can reach even up to 20%, but only 6% would be severe. Among these, only 2-8% are due to arterial lesions. In our series we did not detect any of them.

The differences in the percentages reported are probably due to the definition used in the variable “bleeding complication”. Some authors describe them only when symptomatic or when they generate a fall in hemoglobin levels. Our figures are within the parameters described in the literature even having included all bleeding complications, whether symptomatic or not. This gives strength to our study. In our series, no patient required any extra procedure to control bleeding. Most were self-limited hemobilia. We detect a single patient with hematoma on ultrasound and was completely asymptomatic, no treatment required. 

In a record of the British Society of Interventional Radiology, published by Uberoi et al^24^ they observed 4.5% of bleeding complications, and within them, found a moderate association with a low platelet count. However, the sample size achieved in the group of patients with low platelet count was not enough and did not reach statistical significance. The author did not analyze the correction of hemostasis as a possible risk factor. Choi, Sang Hyun et al^2^, in a retrospective study, analyzed the risk factors for the development of hepatic arterial lesions during PBD and found that the highest RIN 1.5 and lower platelet count to 50000 were associated in the bivariate analysis; however, in multivariate analysis were not statistically significant. They did not assess the correction of coagulation as a possible risk factor either. The only factor that prevailed in the multivariate analysis was the left access. This also matches the description of another retrospective study of Rivera-Sanfeliz, GM et al^20^.

Our results probably differ due to the retrospective nature, the definition and aims of Choi et al. and Rivera-Sanfeliz, GM et al. ^2^ ^,^ ^20^ studies; both, only looked for arterial lesions. In our series, we found no differences even between the access side. However, the sample size is still not enough to draw conclusions on this item. On the other hand, the diameter of the catheter was associated with greater amount of bleeding complications but only on the right side. We believe we need even more cases to draw valid conclusions about this topic. In agreement with other authors^2^ ^,^ ^20^, we found no difference between the number of punctures, drainage type, hematocrit, hemoglobin, bilirubin and albumin values. Nor in platelets and RIN values ​​and this is probably due to the fact that patients who had low levels of coagulation, were the ones corrected with external factors. Notably, there was a statistically significant difference in the values ​​of prothrombin concentration. They were higher in those patients who had bleeding complications, but in both groups above normal threshold values, which subtracts clinical significance.

The sample size is still not enough to analyze each type of correction of hemostasis individually. Maybe, this is the weakness of our study. However, as those who were corrected with vitamin K before the procedure could be compared to patients with normal coagulation (since vitamin K is simply a cofactor for the synthesis of the factors of own clotting), we decided to conduct a separate analysis excluding patients corrected only with vitamin K, and the results did not change. Even without those cases, the percentage of bleeding complications in patients requiring hemostasis correction for a PBD was not greater than in those not requiring it. 

Finally, we found no similar reports in the literature analyzing the correction of hemostasis in PBD. Our study demonstrated that the correction values ​​of hemostasis is safe and is not associated with an increase in bleeding complications. A trend is observed, and it seems to be more complications in the uncorrected group. Sample size should be increased to confirm this trend. 

We believe that our findings are important and applicable to clinical practice when performing a procedure of this type and to decide hemostasis correction when necessary without hesitations, especially if there are other risk factors for bleeding.

## CONCLUSION

The percentage of bleeding complications in patients requiring hemostasis correction for a PBD is not greater than in those who do not.
